# Syphilitic Hydrosarcocele

**Published:** 1875-06

**Authors:** B. J. Preston

**Affiliations:** Rochester, New York


ART. III. *Syphilitic Hydrosarcocle.* By B. J. PRESTON, Rochester, New York.

Among the various forms manifested by syphilitic disease that of Syphilitic Hydrosarcocele is not without considerable interest. It is the object of this paper to relate briefly, the history of an interesting case of this form of syphilis. According to established authority this variety of disease usually attacks the individual after the development of the tertiary form as recognized by excavating ulcerations of the mouth and throat, iritis nodes, etc.

The subject of these remarks, a young man, aged 27 years, applied to me for treatment for a painful swelling affecting his left tibia, which I found upon examination to be in a red, swollen and very painful condition, so much so as to interfere with his ordinary business that of market gardener, and it was upon this account that he was led to seek medical advice. Upon close inquiry I ascertained that he had some four years before contracted a chancre on the penis for which he had been treated, and was, as he supposed, cured.

Believing this to be a true tertiary node, I prescribed a poultice to be applied to the swelling until the inflammatory symptoms had subsided, afterwards local applications of tincture iodine, alternating the iodine and poultices as the circumstances seemed to require. I ordered internally five grain doses of iodide of potassa three times a day combined with vegetable tonics.

Improvement followed rapidly, and although the pain and other inflammatory symptoms soon disappeared he, according to my advice, continued to take the iodide of potassa for about eighteen months, suspended only for intervals of a few weeks.

During the early stage of this treatment he complained of a slight inflammatory symptoms in his left testicle which I directed to be suspended in a suspensory bandage, and heard no more of it until December, 1872.

The patient then complained of his tongue which was nearly perforated by an excavating ulcer nearly two inches in length and very painful. Frequently repeated applications of a strong solution of nitrate of silver put a stop to the destructive process, and the ulcer was healed in a few weeks. It was about this time that he again called my attention to his testicle which, upon a re-examination, I found to present the appearance of an ordinary Hydrocele, and in the course of three or four weeks I operated for the palliative treatment, and drew off about fourteen ounces of a transparent yellowish fluid. After the operation I observed a considerable hardness and some enlargement of the testicle itself, expecting, however, to see him again in a few days I paid but little attention to it. The next interview I had with my patient was about four weeks afterwards when I tapped his scrotum a second time. There was discharged but little fluid, not more than three ounces, and with but small decrease in the size of the tumor.

But upon a third operation the amount of fluid obtained was equal to that at first drawn off. Now for the first time I was able to ascertain the true character of the tumor which, upon deep pressure, seemed to be hard and irregular, there being three distinct nodules. In view of this, and of the previous history of the case, I deemed it necessary that there should be more thorough surgical interference, and therefore called upon Dr. B. L. Hovey, who coincided with me in the opinion that the removal of the organ was necessary. Accordingly on June 7th, 1873, Dr. Hovey, assisted by Dr. C. S. Starr and myself, removed the affected organ.

The weight of the tumor was seventeen ounces. It was ovoid in shape, and smooth and regular on its surface.

Upon laying it open with the scalpel we discovered three distinct sacs containing serum of a slightly yellowish color. The largest of these contained about six ounces of the fluid; the next smaller, three ounces, while the smallest contained only about one ounce. Within the centre of the substance of the testicle itself there was found a deposit of yellow granular matter about the size of a large robin's egg, together with small points of like deposit at the outer edge of the testicle.

A point of interest in the operation is treatment of the cord which was all included in the ligature and without any unpleasant results, such as might be feared from pressure on the spermatic nerve.

This corroborating the experience of Dr. Erichsen may serve as an encouragement to those who have read the cautions and dread the results described in the surgical work of Dr. Syme.

The case progressed favorably. There was but slight constitutional disturbance, the wound suppurating freely, granulation soon filling up the incision.

